# Kaili sour soup in alleviation of hepatic steatosis in rats via lycopene route: an experimental study

**DOI:** 10.1080/07853890.2025.2479585

**Published:** 2025-04-21

**Authors:** Yi Li, Shuo Cong, Rui Chen, Juan Tang, Liqiong Zhai, Yongmei Liu

**Affiliations:** a School of Clinical Laboratory Science, Guizhou Medical University, Guiyang, Guizhou Province, China; b The Third People’s Hospital of Guizhou Province, Laboratory Department, Guiyang, Guizhou Province, China; c Acupuncture and Moxibustion Department, The Affiliated Hospital of Guizhou Medical University, Guiyang, Guizhou, China; d Center for Clinical Laboratories, The Affiliated Hospital of Guizhou Medical University, Guiyang, Guizhou Province, China

**Keywords:** Nonalcoholic fatty liver disease, Kaili sour soup, lipid metabolism, estrogen signaling

## Abstract

**Background:**

Nonalcoholic fatty liver disease (NAFLD) is one of the most prevalent chronic liver diseases, with a range of manifestations, such as hepatic steatosis. Our previous study showed that Kaili Sour Soup (KSS) significantly attenuated hepatic steatosis in rats. This study explored the main components of KSS and the mechanisms by which it exerts its protective effects against NAFLD.

**Methods:**

Twenty-four 6-week-old male Sprague-Dowley (SD) rats were randomly assigned to three treatments: feeding a normal standard diet, a high-fat diet, or a high-fat diet plus gavage KSS. The effects of KSS treatment on hepatic lipid accumulation were assessed using biochemical, histological, and molecular experiments. The amounts of KSS ingredients were measured using biochemical assays. Network pharmacology analyses were performed to identify the hub genes of KSS targets and enriched pathways. CCK-8 assay was used to determine the effect of free fatty acids (FFA), lycopene, and estrogen on HepG2 viability. Quantitative Real-Time polymerase chain reaction (qRT-PCR) and Western blot assays were performed to determine the effect of KSS or lycopene on estrogen signaling and expression of lipid metabolism-related molecules. Statistical analyses were performed using GraphPad Prism and SPSS.

**Results:**

KSS alleviated fat deposition in rat liver tissue and affected the expression of hepatic lipid synthesis, catabolism, and oxidative molecules. Lycopene was identified as the ingredient with the highest amount in KSS. Network pharmacology analyses showed that the hub genes were enriched in the estrogen signaling pathway. Cellular experiments showed that lycopene increased the expression of Estrogen Receptor α (ERα), Carnitine palmitoyltransferase 1 A (*CPT1A*), Peroxisome proliferator-activated receptor α (*PPARα*) (all *p* < 0.01), and Hormone sensitive lipase (*HSL*) (*p* < 0.05), and reduced the expression of lipid metabolism-related factors *1c(SREBP-1c*) (*p* < 0.01), Acetyl-CoA carboxylase 1 (*ACC*) and Lipoprotein lipase (*LPL*) (all *p* < 0.05).

**Conclusions:**

KSS ameliorated abnormal lipid metabolism in patients with NAFLD. Lycopene was the major component of KSS, and it affected estrogen signaling and the expression of lipid metabolism molecules. In short, both KSS and LYC could change lipid metabolism by lowering lipid accumulation and raising lipolysis.

## KEY MESSAGES

1. In this study, the contents of the components of Kaili sour soup, a traditional Chinese fermented food, were examined and analyzed.
2. The effect of Kaili sour soup on hepatic steatosis was shown to be possibly related to the ERα-associated estrogen signaling pathway via a network pharmacology approach.
3. Kaili Sour Soup attenuates hepatic steatosis through its active ingredient, lycopene.

## Introduction

Nonalcoholic fatty liver disease (NAFLD), with a prevalence of 25% and an overall prevalence of nearly 30% in Asia, is one of the most prevalent chronic liver diseases [1]. At least 5% of hepatocytes in patients with NAFLD have steatosis, which can progress to nonalcoholic steatohepatitis, cirrhosis, and hepatocellular carcinoma [2]. Numerous studies have demonstrated that oxidative stress and low-grade inflammatory states are implicated in NAFLD pathogenesis, and emerging evidence suggests that dysregulated lipid metabolism may underlie this disease [3]. Therefore, preventing hepatic lipid accumulation and reducing lipotoxic load could be promising therapeutic strategies against NAFLD [4].

Numerous studies have demonstrated that the deregulation of hepatic lipid metabolism is one of the main causes of NAFLD [5–9]. Imbalances in hepatic lipid metabolism lead to ectopic fat accumulation, which involves excessive exogenous fat intake, enhanced de novo lipogenesis (DNL), and reduced lipolysis and oxidation [10]. These processes are regulated by several molecules involved in lipid metabolism [5–7,11], which could be beneficial in mitigating the progression of NAFLD by modulating dysregulation of lipid metabolism molecule expression. It was confirmed that postmenopausal women have a higher risk of NAFLD than men or premenopausal women [12]. Animal experiments verified dysregulated lipid metabolism in estrogen-deficient mice, which showed an increased accumulation of lipids in the liver compared with normal mice. Hepatic lipid homeostasis in estrogen-deficient mice was improved by supplementation with estradiol (E_2_), and its effect was related to Estrogen Receptor α (ERα) [13].

No drug has been officially approved by the FDA for the treatment of NAFLD. Lifestyle changes and dietary modifications remain the mainstay of improvement in NALFD [14]. Kaili sour soup (KSS) is a traditional fermented food that originated in the Guizhou region of China and is mainly produced by the natural fermentation of tomatoes, red peppers, and salt [15]. Fermented KSS is rich in various minerals, organic acids, lycopene, and capsaicin [16,17]. To date, only a few studies have explored the molecular effects of KSS on NAFLD, and these studies provide limited evidence that KSS has anti-inflammatory and lipid-lowering functions [16,18]. In our study, we sought to first determine the major KSS components and then to employ bioinformatics and network pharmacology approaches to investigate KSS targets and effects in more depth. Additionally, LYC has been confirmed to have antioxidative and anti-inflammatory activities [3]; however, studies investigating the effects of LYC on lipid metabolism are rare [19,20], and the detailed molecular mechanism is not clear.

In this study, the major components of KSS (lycopene, capsaicin, vitamin E, and lactic acid) were examined, and a gene interaction network of the predicted KSS targets was established, in which Toll-like receptor 4 (TLR4), pProtein kinase B (PKB or AKT1), prostaglandin-endoperoxide synthase 2 (PTGS2), and ERα were identified as hub genes. Further functional enrichment analysis suggested that the estrogen signaling pathway may be a critical target of KSS. Furthermore, since LYC is one of the main components of KSS, cellular assays were conducted to explore the effects of LYC on the expression of ERα and lipid metabolism-related genes.

## Materials and methods

### Animal experiments

All animal experiments were approved by the Animal Ethics Committee of Guizhou Medical University and were performed in accordance with the Guide for the Care and Use of Laboratory Animals of China.

A total of 24 six-week-old male Sprague-Dowley (SD) rats weighing 180 ± 20 g were purchased from Changsheng Bio-Technology Inc. (Benxi City, Liaoning, China). All rats were maintained under controlled temperature (23 ± 2 °C) and humidity (60 ± 10%) conditions, with a 12 h light/dark cycle. After adaptive feeding for seven days, the rats were randomly divided into three groups (*n* = 8 for each group): normal diet (ND), high-fat diet (HF), and high-fat diet + KSS(HS) groups [16]. The ND group was fed a normal standard diet (Jiangsu Xietong Pharmaceutical Bio-engineering Inc., Nanjing, Jiangsu, China) containing 80% carbohydrate, 15% protein, and 5% fat, and the HF group was fed a high-fat diet (Jiangsu Xietong Pharmaceutical Bio-engineering Inc.) containing 20% carbohydrate, 20% protein, and 60% fat. The HS group was fed a high-fat diet and gavaged with KSS (1 mL/100 g body weight, Lianghuanzhai, Guizhou, China) once a day for 6 days a week. All the rats had free access to water. Food consumption was recorded daily and the weight of each rat was recorded weekly. After feeding for 12 weeks, all rats were anesthetized with 1% pentobarbital sodium (40 mg/kg) via intraperitoneal injection to collect blood, and liver tissue was collected following the sacrifice of rats. One part of the liver tissue was fixed with 4% paraformaldehyde for pathological histological examination, and the other part was used to assess the effects of KSS on the expression of the lipid metabolism-related key regulatory molecules in HFD-fed rats.

### Pathological–histological examination

The fixed liver tissues were embedded in Optimal Cutting Temperature compound (OCT, Bioss, Beijing, China), and 5-μm sections were cut. Based on the methods described in a previous study [21], the tissue sections were stained with hematoxylin and eosin (HE) or oil red O. Finally, the slides were scanned and photographed under a light microscope (Olympus Corporation, Tokyo, Japan).

### Detection of relevant liver function indicators

To isolate the serum from the blood, the samples were centrifuged at 3000 rpm for 5 min. The levels of aspartate aminotransferase (AST), alanine aminotransferase (ALT), triglycerides (TG), and total cholesterol (TC) in the serum and tissues were measured using a biochemical analyzer (Roche, Basel, Switzerland) and their corresponding commercial kits (Nanjing Institute of Biological Sciences, Nanjing, China) following the manufacturer’s instructions.

### Determination of KSS composition

The composition of KSS, including total acid, lactic acid, vitamin E, calcium, iron, capsaicin, and lycopene contents, was determined by the CMA-qualified China Chengcheng Testing Laboratory in accordance with the relevant national standard procedures, including GB 12456-2021 (total acid), QB/T 5712-2022 (L-lactic acid), GB 5009.82-2016 (vitamin E), GB 5009.92-2016 (calcium), GB 5009.90-2016 (iron), NY/T 1381-2007 (capsaicin), NY/T 1651-2008 (lycopene).

### Construction of KSS-compound component-target gene network

GeneCard and DisGeNET were utilized to obtain the related target genes following the keywords of ‘nonalcoholic fatty liver disease’, ‘fatty liver disease, nonalcoholic’ and ‘nonalcoholic steatohepatitis’. Potential target genes for the major components of KSS were predicted using SEA (http://sea.bkslab.org/), SwissTargetPrediction [19] (http://www.swisstargetprediction.ch/), and SuperPred (https://prediction.charite.de/). After the integration and removal of duplicates, the intersection of NAFLD-related regulatory genes with potential KSS targets was analyzed using the Venn tool. Subsequently, Cytoscape software and the ENRICHR online analysis tool (maayanlab. cloud/Enrichr/) were used for network construction, hub gene analysis, and enrichment analysis. The results of the enrichment analysis were visualized using a bioinformatics online tool (http://www.bioinformatics.com.cn/).

### Cell culture

Human HepG2 cells (American Type Culture Collection, Rockville, MD, USA) were cultured in high-sugar Dulbecco’s modified Eagle’s medium (DMEM, Thermo Fisher Scientific, Massachusetts, USA) supplemented with 10% (v/v) fetal bovine serum (FBS) and 1% (v/v) penicillin/streptomycin (Thermo Fisher Scientific). The medium was changed every 2–3 days, and the cells were passaged regularly.

To induce steatosis in HepG2 cells, 500 μM free fatty acids (FFA) were prepared by mixing oleic acid (15 mM, Macklin, Shanghai, China) with palmitic acid (Macklin) at a ratio of 1:2 and was added to the cells for 24 h in the medium exchange. In the presence or absence of FFA, cells were treated with lycopene (LYC, 5 μM) and estrogen (E_2_, 0.01 μM) for 24 h. The cells with different treatments were then harvested.

### CCK8 experiment

A total of 5 × 10^3^ HepG2 cells per well were cultured in 96-well plates and assigned to three groups: blank group, solvent control group, and treatment group. The following day, the original medium was exchanged with different concentrations of the testing reagents. The following day, cells were incubated with CCK-8 reagent for 1.5 h, and the absorbance was recorded at 450 nm using a microplate reader (Thermo Fisher Scientific, Massachusetts, USA). Three independent experiments were performed.

### RNA isolation and real-time quantitative PCR

Total RNA was extracted from tissue samples using TRIzol reagent (Magen, Guangzhou, China), and cDNA was amplified using RNA reverse transcriptase (Takara, Kyoto, Japan). The real-time quantitative PCR (RT-qPCR) reactions were performed on a LightCycler 480 system (Roche, Basel, Switzerland). All primers were synthesized by Sangon Biotech Co., Ltd.(Shanghai, China) and are listed in Table 1. *ACTB* was used as an internal reference, and the 2^−ΔΔCT^ method was used to analyze the relative expression of related genes [23].

**Table 1.** Primer sequences used for real-time quantitative RCR.

Genes	Forward (5′→3′)	Reverse(5′→3′)
SREBP-1c [22]	GTGGTCTTCCAGAGGCTGAG	GGGTGAGAGCCTTGAGACAG
ACC1 [22]	TACAACGCAGGCATCAGAAG	TGTGCTGCAGGAAGATTGAC
FASN	CTCCCCACTCCAGAACCCAGAC	CCAGCACACCATACGACGTACAG
FAT/CD36	CAGTTCCTACACGACCACCACT	GGACGGATGTCTTCTTCCAGAT
SCD-1	GGACATCGGGGAATTGCTGA	CCGGCTTTCACTCGGATCTT
LPL [6]	GTTACCACCCTGCAGTCCTC	CGCTCACACACTTGGAGAGT
HSL [6]	GGCTGCTATAATTTGCTGTGG	TTTGAAGGAGTTTTGGGAAGAG
PPARα	CCATACTGCTGTATCGTCGCA	CGGGAAGTATTGAAGAGTCGC
CPT1A	CCGTAAAGACCTCTATGCCAACA	CGGACTCATCGTACTCCTGCTT
β-actin	GTGGTCTTCCAGAGGCTGAG	GGGTGAGAGCCTTGAGACAG

SREBP-1c: sterol regulatory element binding protein 1c; ACC1: acetyl-CoA carboxylase 1; FASN: fatty acid synthase; FAT/CD36: fatty acid translocase/cluster of differentiation 36; SCD-1: stearoyl-CoA desaturase 1; LPL: lipoprotein lipase; HSL: hormone sensitive lipase; PPARα: peroxisome proliferator-activated receptor α; CPT1A: carnitine palmitoyl transferase I A

### Western blot

Total protein was isolated from both cell cultures and mice tissue samples using RIPA reagent (Solarbio, Beijing, China), and the concentrations of the isolated proteins were measured using a BCA assay kit (Beyotime, Shanghai, China). Protein samples (20 μg) were separated using 10% SDS-PAGE and transferred to Immobilon-P membranes (Millipore, Bedford, MA, USA). After blocked with 5% skim milk for 2 h, the membranes were incubated with the following primary antibodies, including anti-ERα (21244-1-AP, 1:1000, Proteintech), anti-Carnitine palmitoyltransferase I A (CPT1A) (15184-1-AP, 1:4000, Proteintech), anti- Peroxisome proliferator-activated ­receptor α (PPARα) (15540-1-AP, 1:1000, Proteintech), anti- Acetyl-CoA carboxylase 1(ACC1) (21923-1-AP, 1:4000, Proteintech), anti- Sterol regulatory element binding protein 1c (SREBP-1c) (WL02093, 1:1000, Wanleibio), anti- Lipoprotein lipase (LPL) (bs-1973R, 1:1000, Bioss), anti- Hormone sensitive lipase (HSL) (WL02643, 1:1000, Wanleibio), and anti-β-actin (AC026, 1:50,000, ABclonal) at 4 °C overnight. The membranes were then incubated with the corresponding secondary antibodies (PMK-014-090S, 1:10,000) for 1 h at room temperature. Finally, the protein bands were visualized using a chemiluminescent immunoassay (Solarbio, Beijing, China) and an imaging system (Bio-Rad, CA, USA) and analyzed using ImageJ software (NIH, MD, USA).

### Data analysis

The number of animals used was determined based on previous studies and the results of our preliminary experiments [6,16]. At least three independent experiments were performed for each assay. Statistical analyses were performed using GraphPad Prism 8.3.0 (San Diego, CA, USA) and SPSS 18.0 (SPSS, Inc., Chicago, USA). The Shapiro-Wilk test was used to assess the normality of continuous variables. Normally distributed data are expressed as mean ± standard $\left(\bar{x}\pm s\right)$ deviation. The *t*-test was used to compare the two groups of data with normal distribution; otherwise, the Mann–Whitney test was used, while analysis of variance followed by Tukey or Kruskal–Wallis test was employed to assess significant differences among more than two groups. α = 0.05, and *p* values of <0.05 were considered to be statistically significant.

## Results

### KSS reduced body weight and liver weight in HFD-induced rats

As shown in Table 2, there was no significant difference in the initial body weight among the three groups (*p* > 0.05). After 12 weeks of feeding, the final body weights of the ND, HF, and HS groups were 435.13 ± 23.75 g, 510.63 ± 31.49 g, and 466.88 ± 28.97 g; respectively, indicating that HFD significantly increased the body weight of rats compared to the ND group (*p* < 0.01), whereas KSS gavage evidently reduced the body weight induced by HFD (*p* < 0.05, Table 2). The trend of food intake in the three different groups was opposite to that of final body weight. The liver weight, liver index, and Lee’s index were significantly higher in the HF group than in the ND group (*p* < 0.01), while KSS gavage markedly decreased the liver weight and liver index in the HFD-induced rats (*p* < 0.05) and did not significantly affect the Lee’s index (*p* > 0.05). These results suggest that KSS could reduce the body weight and liver index of HFD-fed rats.

**Table 2.** Effect of KSS on body weight and liver weight in HFD-fed rats.

Parameter	ND group	HF group	HS group
Initial body weight (g)	283.75 ± 10.82	287.00 ± 7.63	281.25 ± 12.02
Final body weight (g)	435.13 ± 23.75	510.63 ± 31.49**	466.88 ± 28.97^#^
Food intake^Δ^(g)	18.08 ± 2.24	11.45 ± 2.14**	15.09 ± 2.72^#^
Liver weight (g)	11.51 ± 1.14	16.12 ± 1.04**	13.55 ± 0.72^##^
Liver index (%)	2.64 ± 0.20	3.16 ± 0.08**	2.91 ± 0.24^#^
Lee’s index	2.96 ± 0.04	3.07 ± 0.05**	3.02 ± 0.06

Data were analyzed by ANOVA followed by Tukey or Kruskal–Wallis test. Results are shown as the mean ± SEM. Liver index= (liver weight/body weight) ×100%; Lee’s index= (weight × 1000)^(1/3)/length (cm). *p < 0.05, **p < 0.01 vs ND group; ^#^*p* < 0.05, ^##^*p* < 0.01 vs HF group. ^Δ^represents the mean food intake of each rat group. *n* = 8.

### Effects of KSS on serum biochemical parameters in HFD-induced rats

Compared with the ND group, the HF group showed ­significant increase in serum TC (7.77 ± 0.19 vs. 2.39 ± 0.35 mmol/L, *p* < 0.01), TG (1.51 ± 0.05 vs. 0.71 ± 0.08 mmol/L, *p* < 0.01), low-density lipoprotein – cholesterol (LDL-C) (1.25 ± 0.03 vs. 1.17 ± 0.03 mmol/L, *p* < 0.01), AST (440.40 ± 40.20 vs. 140.54 ± 17.54 U/L, *p* < 0.01), ALT (125.88 ± 44.40 vs. 57.11 ± 9.88 U/L, *p* < 0.01), liver TC (0.0106 ± 0.0023 vs. 0.0047 ± 0.0015 mmol/g, *p* < 0.01), and liver TG levels (0.0110 ± 0.0026 vs. 0.0052 ± 0.0012 mmol/g, *p* < 0.01), but a significant decrease in high - density ­lipoprotein - cholesterol (HDL-C) levels (0.51 ± 0.05 vs. 1.21 ± 0.08 mmol/L, *p* < 0.01). After 12 weeks of KSS feeding, the levels of serum TC, TG, TC, TG, LDL-C, and AST were significantly reversed by KSS in HFD-induced rats (*p* < 0.05, Table 3).

**Table 3.** Effect of KSS on biochemical analysis in HFD-fed rats.

Parameter	ND group	HF group	HS group
Serum TC (mmol/L)	2.39 ± 0.35	7.77 ± 0.19**	6.34 ± 0.53^##^
Serum TG (mmol/L)	0.71 ± 0.08	1.51 ± 0.05**	1.16 ± 0.16^##^
Liver TC (mmol/g)	0.0047 ± 0.0015	0.0106 ± 0.0023**	0.0071 ± 0.0022^##^
Liver TG (mmol/g)	0.0052 ± 0.0012	0.0110 ± 0.0026**	0.0085 ± 0.0006^#^
Serum LDL-C (mmol/L)	1.17 ± 0.03	1.25 ± 0.03**	1.23 ± 0.03
Serum HDL-C (mmol/L)	1.21 ± 0.08	0.51 ± 0.05**	0.76 ± 0.10^##^
Serum AST (U/L)	140.54 ± 17.54	440.40 ± 40.20**	226.24 ± 80.66^##^
Serum ALT (U/L)	57.11 ± 9.88	125.88 ± 44.40**	99.75 ± 49.19

Results are shown as the mean ± SEM. TC: total cholesterol; TG: triglyceride; LDL-C: low-density lipoprotein cholesterol; HDL-C: high-density lipoprotein cholesterol; ALT: alanine aminotransferase; AST: aspartate aminotransferase

*p < 0.05, **p < 0.01 vs ND group; ^#^*p* < 0.05, ^##^*p* < 0.01 vs HF group. *n* = 8.

### KSS alleviated morphological abnormalities and lipid accumulation in HFD-fed rats

**Figure 1.**
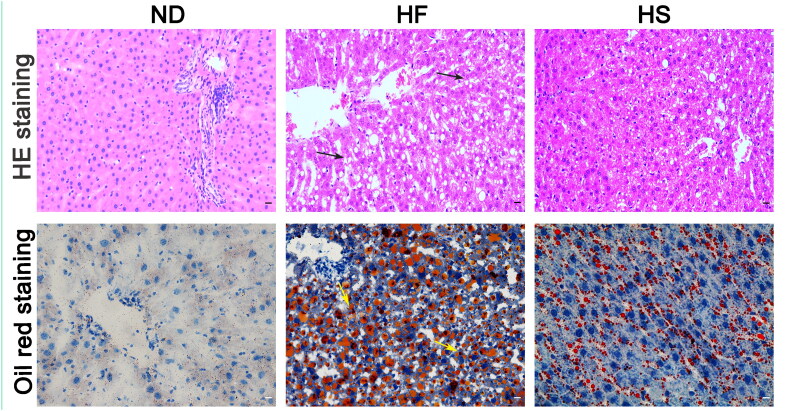
Effect of Kaili sour soup (KSS) on liver histopathology in hepatic steatosis rats. Representative images of liver tissue sections stained with H&E (magnification 200×, upper panel) and oil red O (magnification 200×, lower panel). Black arrows indicate lipid vacuoles, and yellow arrows indicate lipid droplets.

HE staining showed that the liver cells in the ND group were normal with round and centered nuclei, arranged in a regular and dense manner, with the central vein as the center arranged in a cord-like manner. Oil red O staining showed no obvious lipid droplets in the cells (Figure 1). Compared with the ND group, there were dispersed lipid vacuoles, enlarged hepatocytes, disordered hepatocyte cords, compressed liver sinusoids, and more lipid droplets in HFD-fed rats (Figure 1). Additionally, in comparison with the HF group, the rats in the HS group showed improved liver pathology, accompanied by fewer lipid vacuoles and lipid droplets (Figure 1).

### Effects of KSS on the expression of the lipid metabolism-related key regulatory molecules in HFD-fed rats

**Figure 2.**
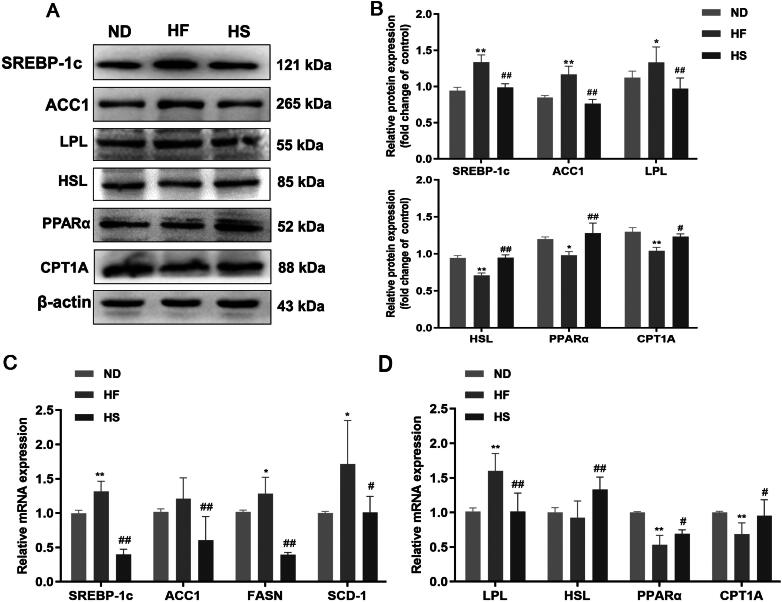
Protein blotting and mRNA analysis of hepatic lipid metabolism molecules in hepatic steatosis rats. (A) Representative bands of protein expression. (B) Relative expression of protein blots of SREBP-1c, ACC1, LPL, HSL, CPT1A, and PPARα in the control (ND) group, model (HF) group, and KSS intervention (HS) group. (C) Gene expression of SREBP-1c, ACC1, FASN, and SCD-1 in the three groups. (D) Gene expression of LPL, HSL, PPAR, and CPT1A in three groups. β-actin was used as an internal control. Data were obtained from at least three independent replicated experiments and analyzed by ANOVA followed by Tukey or Kruskal–Wallis test. Results are expressed $\bar{x}\pm s$ (*n* = 8 per group). Compared with the ND group, *p < 0.05, **p < 0.01; compared with the HF group, ^#^*p* < 0.05, ^##^*p* < 0.01.

Western blotting and RT-qPCR were used to determine the protein and mRNA expression of lipid metabolism-related factors (SREBP-1c), ACC1, Fatty acid synthase (FASN), stearoyl-CoA desaturase 1(SCD-1), LPL, HSL, PPARα, and CPT1A) in HFD-induced rats. Effects of KSS on thCompared with the ND group, the protein expression of SREBP-1c, ACC1, LPL, and HSL was significantly upregulated in the HF group (*p* < 0.05), whereas PPARα and CPT1A were significantly downregulated (*p* < 0.05). However, KSS administration significantly reversed the HFD-induced protein expression (*p* < 0.01, Figure 2(A and B)). In addition, the tendency of SREBP-1c, ACC1, FASN, SCD-1, LPL, HSL, PPARα, and CPT1A in the different groups was similar to their protein expression (Figure 2(C and D)).

### The composition and contents of KSS

It was found that the contents of calcium, lycopene, and total acids in KSS were 111.8 ± 24.99 mg/kg, 95.53 ± 14.66 mg/kg, and 2.55 ± 0.90 g/100 g, which were significantly higher than those in the tomato juice (*p* < 0.05, Table 4). Vitamin E, Fe, and capsaicin were only present in the KSS. Calcium was the primary component, with the highest content in KSS, followed by lycopene and capsaicin (Table 4).

**Table 4.** Composition and content of the KSS.

Component	Tomato juice values	KSS values
Total acids (g/100g)	0.43 ± 0.01	2.55 ± 0.90*
Lactic acid (g/100g)	0.27 ± 0.01	2.23 ± 0.37
Vitamin E (mg/Kg)	–	6.49 ± 3.28
Ca (mg/Kg)	57.73 ± 12.66	111.8 ± 24.99*
Fe (mg/Kg)	–	4.43 ± 2.99
Lycopene (mg/Kg)	48.7 ± 6.35	95.53 ± 14.66*
Capsaicin (mg/Kg)	–	47.87 ± 43.56

The *t*-test was used for the comparison of two groups. Results are shown as $\bar{x}\pm s$ . Kaili Sour Soup (KSS). *n* = 3. *p < 0.05 vs Tomato juice group.

### KSS regulates lipid metabolism through affecting estrogen signaling

**Figure 3.**
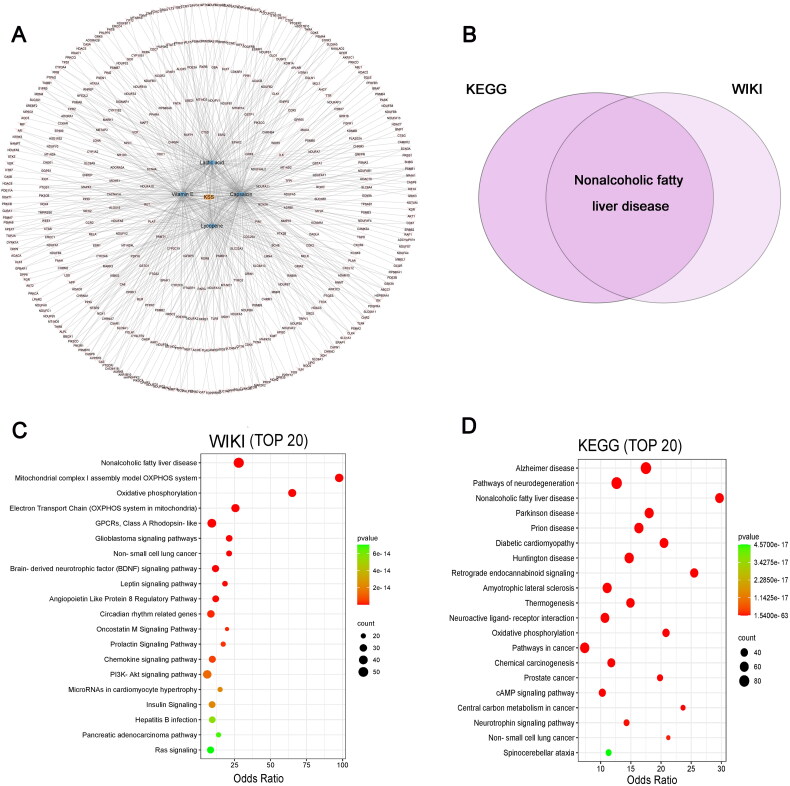
Network pharmacology analysis of KSS target genes and diseases. (A) KSS (yellow)-compound component (blue)-target gene (pink) network. (C) Enrichment analysis of KSS target genes in WIKI databases (top 20). (D) Enrichment analysis of KSS target genes in KEGG databases (top 20). (B) The pathway is present in both KEGG and WIKI enrichment analyses.

Further, network pharmacology analysis was performed to explore the potential mechanisms by which KSS affects lipid metabolism [24]. Lycopene, capsaicin, vitamin E, and lactic acid were selected to be the main components of KSS for subsequent analyses. Next, SEA, SwissTargetPrediction, and target genes of the main components of KSS were predicted using SuperPred, and a total of 428 genes were identified (Figure 3(A)). The enriched WIKI pathways of the 428 genes included ‘nonalcoholic fatty liver disease’, ‘mitochondrial complex I assembly model OXPHOS system’ and ‘oxidative phosphorylation’ (Figure 3(C)); as well as the enriched KEGG pathways contained ‘cAMP signaling pathway’, ‘pathways of neurodegeneration’, ‘nonalcoholic fatty liver disease’, ‘neurotrophin signaling pathway’, ‘thermogenesis’ and ‘oxidative phosphorylation’ (Figure 3(D)). The intersection of the resulting KEGG and WIKI pathways was related to NAFLD (Figure 3(B)).

**Figure 4.**
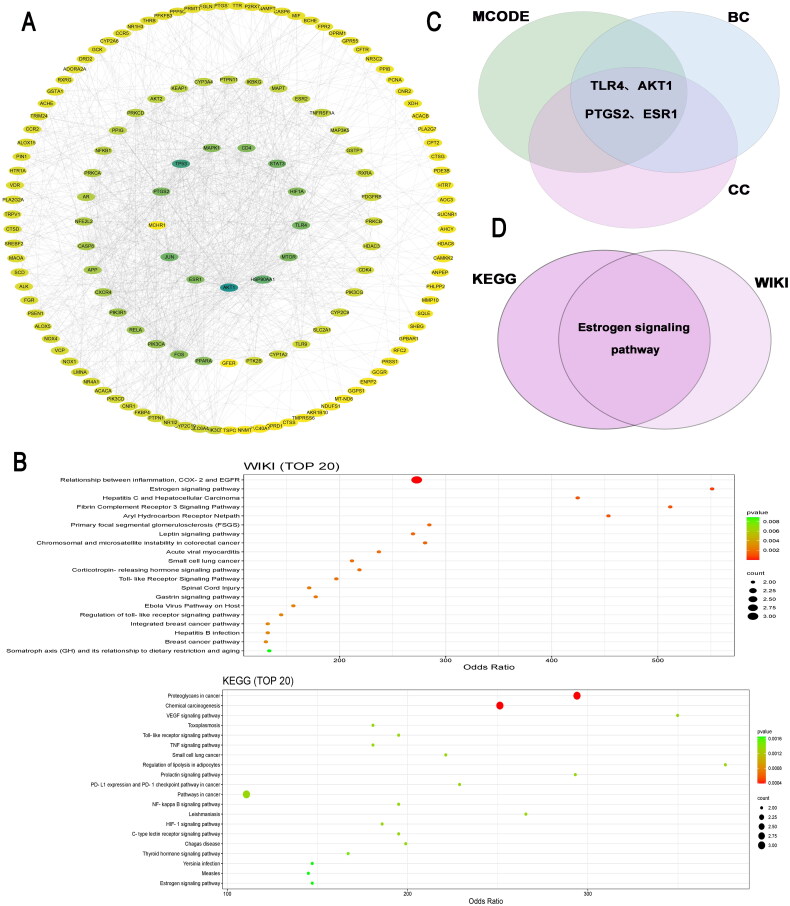
Protein–protein interaction (PPI) network and functional enrichment of NAFLD-associated KSS targets. (A) PPI of NAFLD-associated KSS targets. (C) A Venn plot showing the selection of hub genes using three different methods. (B) Enrichment analysis of NAFLD-associated KSS targets in WIKI and KEGG databases (top 20). (D) Intersection of the resulting pathways in WIKI and KEGG enrichment analyses.

Next, we extracted NAFLD-associated genes and intersected them with 428 KSS-related target genes, and then a gene-gene interaction network was successfully constructed (Figure 4(A)). It was found that TLR4, AKT1, PTGS2, and ERα were the hub genes in the constructed network (Figure 4(C)). After that, functional enrichment of these genes was analyzed, and were enriched in WIKI pathways of ‘relationship between inflammation, Cyclooxygenase-2 (COX-2) and Epidermal growth factor receptor (EGFR)’, ‘estrogen signaling pathway’, ‘leptin signaling pathway’ and ‘fibrin complement receptor 3 signaling pathway’; as well as the KEGG pathways of ‘Vascular endothelial growth factor (VEGF) signaling pathway’, ‘toll-like receptor (TLR) signaling pathway’, ‘TNF signaling pathway’, ‘NF-kappa B signaling pathway’, ‘HIF-1 signaling pathway’ and ‘estrogen signaling pathway’ (Figure 4(B)). Intersection of the resulting KEGG and WIKI pathways resulted in the estrogen signaling pathway (Figure 4(D)).

### Effects of lycopene on the expression of ERα and lipid metabolism related proteins

**Figure 5.**
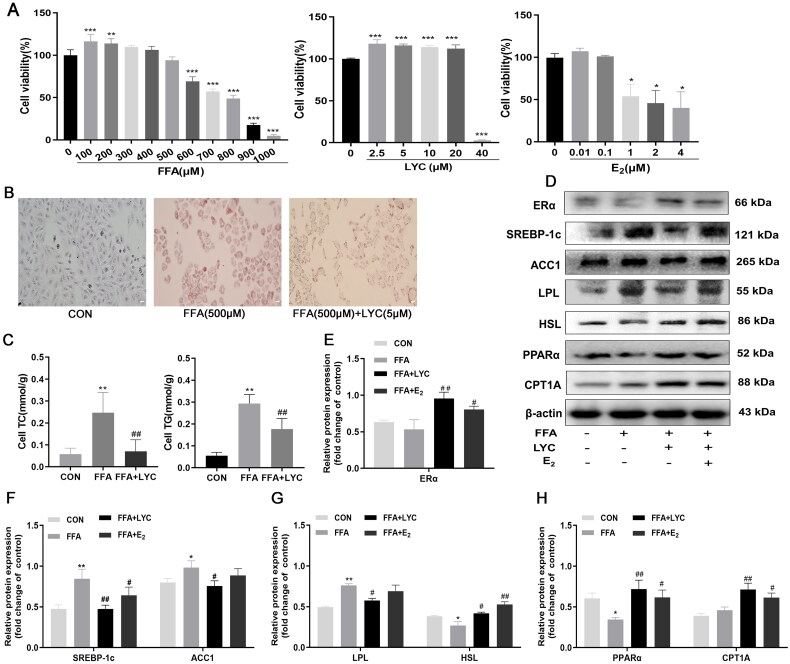
Effect of lycopene (LYC) on HepG2 cells. (A) Cell viability of HepG2 cells induced by different concentrations of FFA, LYC and estrogen for 24 h. (B) Representative images of different groups of treated cells stained with oil red O (200× magnification). (C) TG and TC contents of different groups of treated cells; (D) Representative protein bands of ERα, SREBP-1c, ACC1, LPL, HSL, CPT1A and PPARα. (E)–(H) Quantification of protein blots. Data were obtained from at least three independent experiments and analyzed by ANOVA followed by Tukey or Kruskal-Wallis test. Results are expressed as $\bar{x}\pm s$ (*n* = 3 per group). Compared with the CON group, *p < 0.05, **p < 0.01; compared with the FFA group, ^#^*p* < 0.05, ^##^*p* < 0.01.

The concentrations of FFA, LYC, and E_2_ in the cellular assays were determined in preliminary experiments (Figure 5(A and B)). Cell viability significantly decreased when cells were treated with FFA ≥ 600 μM, LYC ≥ 40 μM, and E_2_ ≥ 1 μM compared to the control. Oil red O staining results showed that compared with the control, 500 μM FFA induced steatosis in HepG2 cells, whereas 5 μM LYC treatment reduced the size of the deposited lipid droplets (Figure 5(B)). Therefore, 500 μM FFA, 5 μM LYC and 0.1 μM E_2_ were chosen as working concentrations for subsequent experiments. FFA treatment significantly increased TG and TC levels (*p* < 0.05), whereas LYC treatment decreased TG and their levels (*p* < 0.05, Figure 5(C)). Next, the expression of ERα and lipid metabolism-related proteins was examined using western blotting. In comparison with the FFA-induced cells, LYC treatment increased the protein levels of ERα, HSL, PPARα and CPT1A, and significantly reduced the levels of SREBP-1c, ACC, and LPL (*p* < 0.05, Figure 5(D–H)). Additionally, E_2_ treatment significantly increased the levels of ERα, HSL, PPARα, and CPT1A (*p* < 0.05), and decreased the levels of SREBP-1, ACC1, and LPL in FFA-treated cells, although to a lesser extent (Figure 5(D–H)).

## Discussion

NAFLD is a detrimental chronic liver disease that can progress to more severe liver illnesses, including nonalcoholic steatohepatitis, cirrhosis, and hepatocellular carcinoma [5]. Currently, there is no curative medication for NAFLD, and several studies and previous survey showed that KSS was protective against this disease [16,18]. A survey conducted in our laboratory indicated that fat metabolism is more efficient among individuals from the local population who like Kaili Sour Soup compared to those who do not [16,25]. It is necessary to note, nonetheless, that our team did not perform clinical population studies following the gathering of survey data, in contrast to earlier studies [26,27]; hence, more clinical trials are required for direct evaluation and verification.

Through network pharmacology analysis to explain the possible mechanisms [24], the results showed that 428 target genes of the major KSS components were identified. Further gene interaction network construction and bioinformatic analyses identified TLR4, AKT1, PTGS2, and ERα as key targets mediating the protective effects of KSS. TLR4 recognizes pathogen-associated molecular patterns expressed on infectious agents, mediates the production of proinflammatory cytokines, and has been shown to be a key inducer of hepatic inflammation in NAFLD [28,29]. PTGS2 is one of the two isozymes of prostaglandin-endoperoxide synthase and has been used as a molecular marker for iron death [30,31]. Oxidative cellular damage caused by iron may be associated with chronic liver diseases, including nonalcoholic steatohepatitis (NASH) and HCC, and lipid peroxidation induced by reactive oxygen species is considered the underlying pathological mechanism [32]. However, the effect of PTGS2 on NAFLD remains unexplored, and our results indicate that it may also have a key pathogenic role in NAFLD. ERα is a key receptor that mediates estrogen signaling and regulates the expression of lipid synthesis genes [33]. Several studies have suggested its indispensable role in maintaining liver homeostasis [34,35]. Our results corroborate these findings and imply that ERα may have a pathogenic role in NAFLD, which requires further investigation.

Functional enrichment analyses of the KSS target genes revealed the critical role of inflammatory, hypoxia sensing, angiogenic, and estrogen signaling pathways in mediating the protective effects of KSS against NAFLD, including the signaling pathways of TLR, TNF, NF-kappa B, hypoxia-inducible factor-1 (HIF-1), vascular endothelial growth factor (VEGF), and E_2_. Inflammatory events in adipose tissue have been shown to drive NAFLD pathogenesis [4], and our results indicated that KSS may target key proinflammatory cytokines and receptors (TLR, TNF, NF-kappa B) to prevent NAFLD progression. HIF-2α upregulation promotes lipid synthesis through the PI3K-AKT-mTOR pathway [36], and our results suggest that KSS may lower lipid accumulation through HIF-1 signaling. Angiogenesis is mainly driven by the proangiogenic cytokine VEGF, and elevated serum VEGF levels in NAFLD patients promote hepatic inflammation and fibrosis [37]. Estrogen signaling pathways play a protective role in NAFLD [38]. Our results indicate that KSS may protect against NAFLD by modulating these pathways.

LYC had the highest amount of KSS component, and cellular assays showed that LYC treatment significantly affected the expression of lipid metabolism-related genes, indicating that it regulates hepatic lipid metabolism and prevents lipid accumulation in the liver. SREBP-1c is one of a key transcription factor regulating de novo lipogenesis (DNL) [39]. ACC is the key enzyme that converts malonyl-CoA to acetyl-CoA, thereby controlling the balance between DNL and fatty acid oxidation [40]. LPL and HSL are rate-limiting enzymes that hydrolyze triacylglyceride (TAG) [20,41]. CPT1A encodes a transferase in the outer mitochondrial membrane that regulates the entry of fatty acids into the mitochondria [39]. The ligand-activated transcription factor PPARα is a critical regulator of lipid catabolism [39]. WB blotting demonstrated that LYC significantly reduced the cellular levels of SREBP-1c, ACC1, and LPL and increased the levels of HSL, CPT1A, and PPARα in FFA-treated cells. Our results are consistent with those of previous studies that explored the effect of LYC on the expression of SREBP-1c, ACC1, HSL, CPT1A, and PPARα in rats and hens fed a high-fat diet [19,20]. Additionally, we observed the effects of E_2_ on the expression of lipid metabolism-related genes, which is in line with previous studies [42]. Network pharmacology analysis suggested that estrogen signaling may be a major target of KSS; therefore, we examined the effect of LYC on the expression of ERα. Our results demonstrated that LYC significantly increased the expression of ERα, implying that it may augment estrogen signaling. However, we did not directly examine the estrogenic bioresponses induced by LYC, such as the effect of LYC treatment on the expression of estrogen-responsive genes, and relatively, its clinical utility has also yet to be demonstrated [43]. Future studies are needed to verify and directly assess the specific effects of LYC on the estrogen signaling pathway.

## Conclusion

In summary, our study revealed that KSS could slow the progression of NAFLD through its functional component lycopene, and the underlying mechanism was possibly related to its regulation of lipid metabolism molecules and the estrogen signaling pathway.

## Supplementary Material

supplementary material.pdf

## Data Availability

All data generated or analyzed during this study are included in this article. Further inquiries can be directed to the corresponding authors.

## Additional statement

The manuscript adhered to ARRIVE guidelines.
